# Detection of Congenital Syphilis via Digital PCR and Next-Generation Sequencing, Colombia

**DOI:** 10.3201/eid3208.251737

**Published:** 2026-08

**Authors:** Ana M. Bossa-Castro, Narda M. Olarte Escobar, Ismael Alberto Valderrama Márquez, Deisy Abril, Luis Fuentes, Zayda Lorena Corredor-Rozo, Ruth Liliana López Cruz, Sharon Hassbleidy Ochoa Ramírez, José Alejandro Mojica Madera, Pedro José Ramos Cabrera, Jorge Eliecer Castellanos Corredor, Nidia Aydee Garay Bernal, Ana María Gómez Puentes, Carlos Alberto Morales Pertuz, Javier Escobar-Pérez

**Affiliations:** Universidad El Bosque, Vicerrectoría de Investigaciones, Bacterial Molecular Genetics Laboratory, Bogotá, Colombia (A.M. Bossa-Castro, D. Abril, L. Fuentes, Z.L. Corredor-Rozo, A.M. Gómez Puentes, J. Escobar-Pérez); Subred Integrada de Servicios de Salud Sur E.S.E., Bogotá (N.M. Olarte Escobar, I.A. Valderrama Márquez, R.L. López Cruz, S.H. Ochoa Ramírez, J.A. Mojica Madera, P.J. Ramos Cabrera, J.E. Castellanos Corredor, N.A. Garay Bernal, C.A. Morales Pertuz)

**Keywords:** Syphilis, congenital syphilis, Treponema pallidum, digital PCR, next-generation sequencing, bacteria, Colombia

## Abstract

We describe congenital syphilis in a newborn whose mother had secondary syphilis diagnosed during pregnancy and received a single penicillin dose. Molecular methods detected an extremely low *Treponema pallidum* load in the mother but higher load in the neonate. Our findings support using molecular methods for detecting vertical *T. pallidum* transmission.

Syphilis is an infectious bacterial disease caused by *Treponema pallidum* subspecies *pallidum*. Mother-to-child transmission, known as congenital syphilis (CS), can result in severe adverse outcomes, including fetal and neonatal death, stillbirth, and preterm or low-birthweight births (*1*). Gestational syphilis and CS diagnoses rely on a combination of treponemal and nontreponemal serologic tests (*1*–*3*). However, those methods can yield false-negative results, particularly in early disease stages, because of low initial bacterial load, prozone effect (primarily in secondary syphilis), or co-occurrence with other diseases (*2*). We describe a case of vertical neonatal *T. pallidum* transmission in Bogotá, Colombia, and use of enhanced molecular methods for CS diagnosis.

## The Study

A 27-year-old pregnant woman was admitted to Meissen Hospital in Bogotá for threatened preterm labor at 32-weeks’ gestation on the basis of third-trimester ultrasound. She had a history of 3 previous pregnancies, including a stillbirth. She reported receiving no prenatal care during this pregnancy and a previous syphilis diagnosis with a nontreponemal Venereal Disease Research Laboratory (VDRL) titer of 1:4 but was unsure whether she received treatment. Physical examination revealed widespread, well-defined desquamative skin lesions, initially suggestive of psoriasis. At admission, a positive treponemal test and a VDRL titer of 1:2 led to a diagnosis of latent syphilis of unknown duration. She was prescribed 3 doses of 2,400,000 IU of benzathine penicillin, administered weekly. However, she received only the first dose because she requested a voluntary discharge. Thirty-six days later, she was re-admitted in the expulsive stage of labor but had not received further penicillin doses. Urine toxicology was positive for cocaine and cannabinoids. Her VDRL titer remained at 1:2 and her benzathine penicillin regimen was reinitiated. She again requested voluntary discharge before completing treatment (Appendix).

A female neonate was born via vaginal delivery at 36-weeks’ gestation. The newborn had low birthweight, mild cyanosis, respiratory distress syndrome, and secondary apnea. Initial laboratory findings included thrombocytopenia and indirect hyperbilirubinemia (Table). Results of urinalysis, long-bone radiography, echocardiography, brain ultrasound, and ophthalmologic evaluation were unremarkable; chest radiographs showed no pneumonia. Blood culture results were negative. VDRL tests of blood and cerebrospinal fluid (CSF) were nonreactive (Figure). Maternal and neonatal VDRL testing was performed at the same facility using identical kits and standardized laboratory protocols.

**Table:** Newborn clinical test results for detection of congenital syphilis via digital PCR and next-generation sequencing, Colombia*

Parameter	Patient values	CS range (reference)
Blood		
Leukocytes, cells/µL	11,090	>35,000 (*3*,*4*)
Platelets, cells/µL	137,000	<150,000 (*3*,*4*)
AST, U/L	26	>34.46 (*3*,*4*)
ALT, U/L	<7	>12.35 (*3*,*4*)
Total bilirubin, µmol/L	85.33	NA†
Direct bilirubin, µmol/L	13.33	>20% of total bilirubin (*3*,*4*)
Cerebrospinal fluid		
Leukocytes, cells/mm^3^	3	<15 (*3*,*5*)
Erythrocytes, cells/mm^3^	5 (100% fresh)	NA
Proteins	92.2	Premature newborn, <170 (*3*,*5*); full-term newborn <30 days of age, <120 (*3*,*5*)
Glucose, mg/dL	58	NA
VDRL test	Nonreactive	Reactive

*ALT, alanine transaminase; AST, aspartate aminotransferase; CS, congenital syphilis; NA, not applicable; VDRL, venereal disease research laboratory. †No parameter suggestive of CS has been established.

**Figure:**
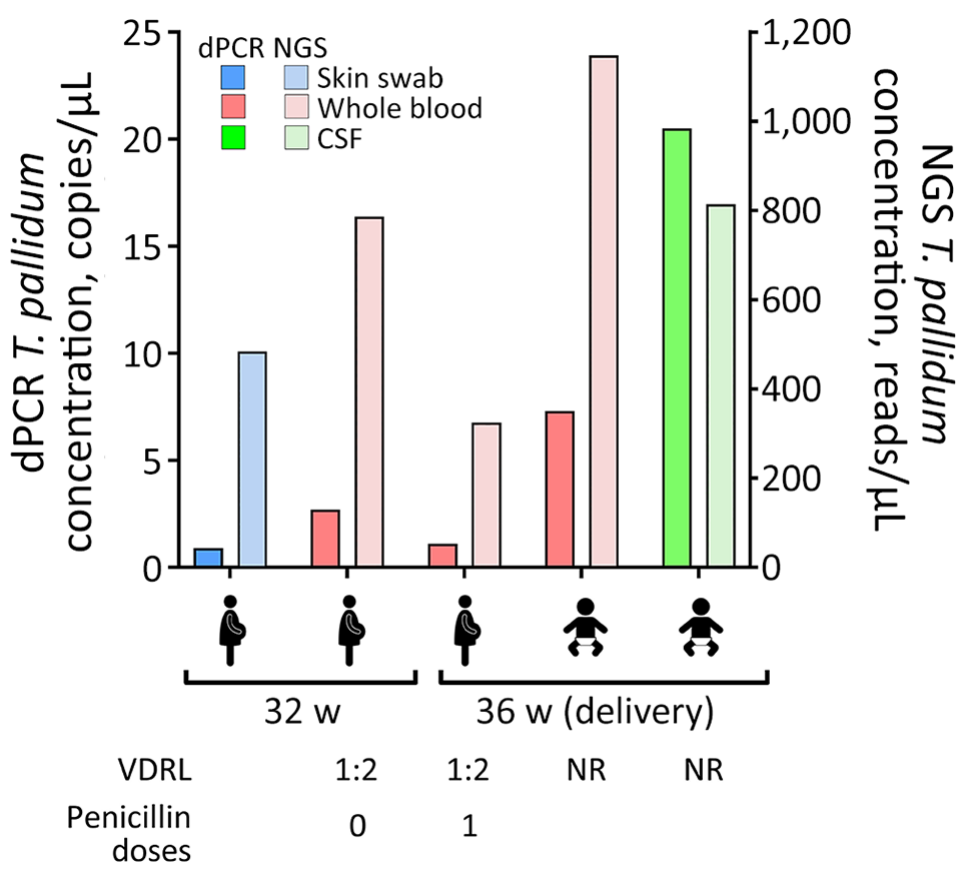
Comparison of testing results for detection of congenital syphilis via dPCR and NGS, Colombia. The *Treponema pallidum tp0547* gene concentration was calculated by the dPCR platform software, and NGS sequencing was performed on the MinION Mk1B (Oxford Nanopore Technologies, https://www.nanoporetech.com). The VDRL results at each time of sampling and number of confirmed penicillin doses in the mother are listed below the graph. CSF, cerebrospinal fluid; dPCR, digital PCR; NGS, next-generation sequencing; NR, not reactive; VDRL, venereal disease research laboratory.

Because of the mother’s high-risk profile (lack of prenatal care, incomplete syphilis treatment, a history of stillbirth, and psychoactive substance use), the newborn was empirically treated with a 10-day course of crystalline penicillin for possible CS. The nonreactive serologic results suggested CS was less likely, according to established Colombia diagnostic criteria (*4*) and US Centers for Disease Control and Prevention guidelines (*2*). We presumed the newborn’s VDRL results were false-negative because of prozone phenomenon (*6*), although that was not confirmed by performing further laboratory testing, such as serial dilutions.

Digital PCR (dPCR) and next-generation sequencing (NGS) assays directly detect DNA and have the potential to diagnose diseases caused by low-abundance pathogens (*7**–**9*), such as *T. pallidum*. During the mother’s initial hospitalization, whole-blood and skin swab samples were collected for molecular analysis. At delivery, a second maternal whole-blood sample was collected, as were neonatal whole-blood and CSF samples. We extracted total DNA from all samples and amplified the *T. pallidum* *tp0574* (*Tpp47*) gene by using dPCR and an in-house targeted NGS assay, then sequenced on a MinION Mk1B (Oxford Nanopore Technologies, https://nanoporetech.com). The maternal whole-blood sample collected at 32-weeks’ gestation had a *T. pallidum* load of 2.7 copies/µL (Figure), which decreased to 1.1 copies/µL after the single benzathine penicillin dose. The VDRL titer remained stable. The premature newborn had a *T. pallidum* load of 7.3 copies/µL in the whole-blood sample and 20.5 copies/µL in CSF, despite nonreactive neonatal VDRL tests (Figure). 

The NGS assay confirmed *T. pallidum* in all samples, the *T. pallidum* load reduction in the mother at delivery, the higher bacterial load in the newborn, and bacterial DNA (511 reads/µL) from the maternal skin swab sample (Figure). Thus, the maternal diagnosis changed from latent syphilis to secondary syphilis, and this case was reclassified as confirmed proven or highly probable CS, highlighting limitations of serologic tests in some high-risk scenarios.

CS incidence is resurging worldwide despite available prenatal screening and timely treatment (*6*). The current diagnostic algorithm using serologic tests has challenges, such as false-positive results because of transplacental passage of maternal IgG, or false-negative results, as in this case. Our findings show the advantages of nucleic acid amplification tests (NAATs) in high-risk scenarios. No commercially approved NAATs are available for syphilis, even for lesions (*6*). However, NAATs could be complementary diagnostic tools in cases of suspected CS, specifically for neonatal blood and CSF samples (*6*), and new technologies, such as dPCR and NGS-based assays, could be used to detect acute or probable chronic active syphilis (*1*).

In vitro studies of *T. pallidum* cultures and in vivo studies in C4D guinea pigs have demonstrated infectivity at low concentrations (*10*,*11*). The molecular techniques we used revealed mother-to-child transmission, even though *T. pallidum* load was low in the mother’s bloodstream. Test results for the mother’s blood after a dose of penicillin showed decreased bacterial loads, but the newborn’s blood and CSF showed elevated values (Figure). Of note, the newborn’s CSF was VDRL-nonreactive and lacked changes in cellularity or protein levels suggestive of neurosyphilis. Although 40%–60% of infants with CS show CSF abnormalities, negative CSF VDRL or normal cell counts do not rule out *T. pallidum* infection or the potential for developing neurosyphilis later (*12*), and such infections would be missed by currently available diagnostic tools.

Molecular confirmation of *T. pallidum* in the mother’s skin lesions, initially suspected to be psoriasis, warranted reclassification as secondary syphilis rather than latent syphilis of unknown duration, as it was initially staged. That finding underscores the need to include syphilis in the differential diagnosis of atypical dermatologic conditions. Furthermore, it highlights the value of molecular testing in refining the diagnosis within a complex clinical setting and in adjusting treatment.

In this case, secondary syphilis diagnosis is particularly noteworthy because, according to Colombia treatment protocols (*3*) and US guidelines (*2*), the single benzathine penicillin dose the patient received 36 days before delivery should have prevented CS. However, dPCR and NGS results indicated vertical transmission. The effectiveness of benzathine penicillin, the only known effective antimicrobial drug for treating fetal infection, depends on multiple factors, including the stage of maternal infection, the number of spirochetes in the blood, severity of fetal infection, timing of treatment initiation, and penicillin levels in fetal tissues (*13*). 

Because of the mother’s high-risk clinical profile, the newborn was empirically treated with a 10-day course of crystalline penicillin for possible CS. The mother had no evidence of reinfection or relapse; however, dPCR and NGS results indicated vertical *T. pallidum* transmission.

## Conclusions

We present compelling evidence of *T. pallidum* vertical transmission despite an extremely low maternal bacterial load. Our findings underscore substantial serologic test limitations and the enhanced detection capabilities of dPCR and NGS. Our results also highlight the need for comprehensive prenatal screening and reassessment of existing standards of care. Diagnosing CS after the administration of a dose of penicillin to pregnant women 30 days before delivery suggests the need for further research. We advocate for integrating highly sensitive NAATs into the diagnostic algorithm for high-risk mothers and their newborns to enable more accurate and timely CS diagnoses, potentially improving clinical outcomes and reducing the incidence of missed cases.

Appendix Additional information for detection of congenital syphilis via digital PCR and next-generation sequencing, Colombia.
