# Supplementary material for: Detection of Congenital Syphilis via Digital PCR and Next-Generation Sequencing, Colombia
Source: Emerg Infect Dis. 2026 Aug;32(8):1341–4. doi: 10.3201/eid3208.251737 (PMC13426889; doi:10.3201/eid3208.251737)
Supplement: Appendix — Additional information for detection of congenital syphilis via digital PCR and next-generation sequencing, Colombia. [file 25-1737-Techapp-s1.pdf]

Article DOI: https://doi.org/10.3201/eid3208.251737

EID cannot ensure accessibility for supplementary materials supplied by authors. Readers who have difficulty accessing supplementary content should contact the authors for assistance.

Detection of Congenital Syphilis via Digital PCR and Next-Generation Sequencing, Colombia

Appendix

Case Presentation

The mother provided confused and incomplete information regarding her history, she did not know her last menstrual period date. She was hospitalized with a diagnosis of a 32-week pregnancy (based on a third-trimester ultrasound), confirmed threatened preterm labor by cervicometry, and low maternal weight with a body mass index of 18. Upon physical examination, she presented with bilateral hypertrophy of the parotid glands that were painful to the touch, which was considered a viral parotitis. The patient requested a voluntary discharge, and the dermatology evaluation for the skin lesions was pending. Subsequently, a telephone and home search was conducted to ensure the application of the two remaining doses of penicillin, but the patient was not found. She was re-admitted 36 days later and the skin lesions were again identified. Her VDRL titer remained at 1:2. Other STORCH studies (HIV, hepatitis B, toxoplasma) were negative. Due to suspected chorioamnionitis, she was prescribed antimicrobial treatment. She again requested a voluntary discharge without completing the initiated treatment or the requested dermatology evaluation.

A female neonate was born via vaginal delivery at 36 weeks of gestational age (determined by Ballard score), with neonatal adaptation APGAR 8/10- 9/10- 10/10, low birthweight of 2,420 g. During labor, hyperthermic amniotic fluid was noted. The neonate was hospitalized with low-flow oxygen via nasal cannula and presumptive diagnosis of infection due to maternal chorioamnionitis. The neonate's nonreactive VDRL was interpreted as a potential false-negative related to prozone phenomenon (*1*).

Molecular Studies

DNA extractions from the clinical samples were carried out using the DNeasy Blood & Tissue Kit (Qiagen, Hilden, Germany) following the manufacturer's recommendations. A double-stranded oligo of a 369 bp fragment of the *tp0574* gene (GenBank: QCQ80232.1) named gBlock:*Tpp47* was synthesized by Integrated DNA Technologies (Coralville, Iowa, USA) to use as positive control DNA in the dPCR assay. Nucleic acid amplification tests (NAATs) amplifying the *Tpp47* gene are highly specific (98%–100%) and have been performed on different types of clinical samples (*1–3*).

dPCR based on nanoplates was carried out using the QIAcuity ONE PCR system (Qiagen, Hilden, Germany). Each reaction was brought to a final volume of 40 µl containing 1X QIAcuity Probe Mastermix (Qiagen, Hilden, Germany), 0.3 µM of each primer, and 0.4 µM of probe (*4*). The following temperature cycles were used: initial denaturation at 95°C for 2 minutes, 40 amplification cycles with 15 seconds of denaturation at 95°C, followed by 30 seconds of annealing at 56°C. After amplification, the fluorescence of the nanowells was detected (default imaging conditions). According to the results obtained from the standarization of the platform, 12 µl of DNA was used for the maternal blood analyses and 22 µl of DNA for the remaining samples. DNA from clinical samples negative for syphilis and reagent controls were used as negative controls.

Based on a comparative genomics analysis of the *T. pallidum* genomes publicly available, a 723 bp region of interest was selected for Oxford Nanopore Technologies (ONT) sequencing assay (https://nanoporetech.com). A double-stranded oligo of this fragment (gBlock:*Tpsp*) was synthesized by Integrated DNA Technologies (Coralville, Iowa, USA) to use as positive control DNA. Primer-barcode pairs were designed according to the barcode sequences reported by Lieberman et al., 2022 (*5*). Barcode sequences were added to the 5′ ends of our sense and antisense primers, which were subsequently synthesized by Macrogen Inc. (Seoul, Republic of Korea). The amplification of this region was standardized by conventional PCR. A reaction with a final volume of 20 µL was carried out, which contained the DreamTaq DNA Polymerase enzyme (Thermo Scientific) at a final concentration of 1.25 U, 0.4 µM of each of the barcode-primers, 0.2 µM of the dNTP mix. The amplifications were carried out using the T100 Thermal Cycler (Bio-Rad), using the following protocol: an initial denaturation of 3 minutes at 95°C,

followed by 30 cycles of 30 seconds at 95°C of denaturation, 30 seconds at 62°C of annealing and 72°C at 40 seconds of elongation; lastly, a final elongation of 5 minutes at 72°C.

The sequencing library was made using the protocol proposed by the native barcoding kit 24 v14 (SQK-NBD114.24) from ONT. For these, 1 µl of the PCR product was taken from each of the combinations and all amplicons from different samples were mixed to form an amplification pool. A positive control pool (gBlock) and a sample pool were obtained, and they were labeled with different ONT barcodes to bioinformatically differentiate the sample and control readings. DNA from clinical samples negative for syphilis, DNA from other bacterial pathogens (*E. coli* and *P. aeruginosa*) as well as reagent controls were used as negative controls. Approximately 800 ng of library were assembled in the R.10.4.1 cell and sequenced using the MinION Mk1B device.

The bioinformatic analysis was divided into three basic parts, basecalling, trimming, and alignment. The base-calling was done with V.1.1 of Dorado tool (https://github.com/nanoporetech/dorado), using "sup" model, "no-trim" parameter and a minimum quality of 20. With the fastq generated, trimming was done with Cutadapt tool (*6*), using two fasta files with the custom barcodes used, first assigning with the forward barcodes, using an overlaping of 10, and an error rate of 0.25. A second trimming was done with an overlaping of 10 and an error rate of 0.15, using the reverse barcodes (*6*). With all the sample assignments finished, alignment was done with minimap2 (*7*) using "*Treponema pallidum* subsp. *pallidum* SS14" as reference (NCBI:txid455434). Read counts aligned per sample were used for comparison between them and with dPCR results.

The 16S rRNA gene V3-V4 region was sequenced from the maternal skin swab DNA using NovaSeq (Illumina). Reads were analyzed in QIIME2-DADA2 (*8*,*9*), and taxonomic identification was performed using SILVA 138.1 database (*10*,*11*). One Amplicon Sequence Variant (ASV) related to the genus *Treponema* was identified (0.19%). Species-level identification of the ASV was not possible because only one out of nine variable regions of the 16S rRNA gene was sequenced.

References

1. Papp JR, Park IU, Fakile Y, Pereira L, Pillay A, Bolan GA. CDC laboratory recommendations for syphilis testing, United States, 2024. MMWR Recomm Rep. 2024;73:1–32. PubMed https://doi.org/10.15585/mmwr.rr7301a1

2. Salle R, Mayslich C, Grange PA, Leducq V, Ollagnier G, Heller U, et al. Specific detection of *Treponema pallidum* in clinical samples: validation of a qPCR assay combining two genomic targets. Sex Transm Infect. 2023;99:91–6. PubMed

3. Meng Y, Yang L, Fu Y, Li S, Hamal K, Liu D. Detection of *Treponema pallidum tpp47* DNA in clinical samples of syphilis patients. Eur J Med Res. 2025;30:873. PubMed https://doi.org/10.1186/s40001-025-03148-4

4. Li M, Lv Y, Cui D, Xu Y, Lin M, Zhang X, et al. Development and clinical validation of a one-step pentaplex real-time reverse transcription PCR assay for detection of hepatitis virus B, C, E, *Treponema pallidum*, and a human housekeeping gene. BMC Infect Dis. 2023;23:358. PubMed https://doi.org/10.1186/s12879-023-08240-w

5. Lieberman NAP, Lin MJ, Xie H, Shrestha L, Nguyen T, Huang ML, et al. *Treponema pallidum* genome sequencing from six continents reveals variability in vaccine candidate genes and dominance of Nichols clade strains in Madagascar. PLoS Negl Trop Dis. 2021;15:e0010063. PubMed https://doi.org/10.1371/journal.pntd.0010063

6. Martin M. Cutadapt removes adapter sequences from high-throughput sequencing reads. EMBnet J. 2011;17:10. https://doi.org/10.14806/ej.17.1.200

7. Li H. Minimap2: pairwise alignment for nucleotide sequences. Bioinformatics. 2018;34:3094–100. PubMed https://doi.org/10.1093/bioinformatics/bty191

8. Bolyen E, Rideout JR, Dillon MR, Bokulich NA, Abnet CC, Al-Ghalith GA, et al. Reproducible, interactive, scalable and extensible microbiome data science using QIIME 2. Nat Biotechnol. 2019;37:852–7. PubMed https://doi.org/10.1038/s41587-019-0209-9

9. Callahan BJ, McMurdie PJ, Rosen MJ, Han AW, Johnson AJ, Holmes SP. DADA2: High-resolution sample inference from Illumina amplicon data. Nat Methods. 2016;13:581–3. PubMed https://doi.org/10.1038/nmeth.3869

10. Quast C, Pruesse E, Yilmaz P, Gerken J, Schweer T, Yarza P, et al. The SILVA ribosomal RNA gene database project: improved data processing and web-based tools. Nucleic Acids Res. 2012;41:D590–6. PubMed https://doi.org/10.1093/nar/gks1219

11. Yilmaz P, Parfrey LW, Yarza P, Gerken J, Pruesse E, Quast C, et al. The SILVA and “All-species Living Tree Project (LTP)” taxonomic frameworks. Nucleic Acids Res. 2014;42:D643–8. PubMed https://doi.org/10.1093/nar/gkt1209
